# Extracorporeal Membrane Oxygenation (VA-ECMO) in Management of Cardiogenic Shock

**DOI:** 10.3390/jcm12175576

**Published:** 2023-08-26

**Authors:** Klaudia J. Koziol, Ameesh Isath, Shiavax Rao, Vasiliki Gregory, Suguru Ohira, Sean Van Diepen, Roberto Lorusso, Chayakrit Krittanawong

**Affiliations:** 1 School of Medicine, New York Medical College and Westchester Medical Center, Valhalla, NY 10595, USA; 2 Department of Cardiology, Westchester Medical Center, Valhalla, NY 10595, USA; 3 Department of Medicine, MedStar Union Memorial Hospital, Baltimore, MD 21218, USA; 4 Division of Cardiothoracic Surgery, New York Medical College and Westchester Medical Center, Valhalla, NY 10595, USA; 5 Division of Cardiology and Critical Care, University of Alberta, Edmonton, AB T6G 2R3, Canada; 6 Cardio-Thoracic Surgery Department, Heart & Vascular Centre, Maastricht University Medical Centre, Cardiovascular Research Institute Maastricht, 6202 AZ Maastricht, The Netherlands; 7 Cardiology Division, NYU Langone Health and NYU School of Medicine, New York, NY 10016, USA

**Keywords:** extracorporeal membrane oxygenation (ECMO), venoarterial, cardiogenic shock (CS), mechanical circulatory support (MCS), left ventricle (LV)

## Abstract

Cardiogenic shock is a critical condition of low cardiac output resulting in insufficient systemic perfusion and end-organ dysfunction. Though significant advances have been achieved in reperfusion therapy and mechanical circulatory support, cardiogenic shock continues to be a life-threatening condition associated with a high rate of complications and excessively high patient mortality, reported to be between 35% and 50%. Extracorporeal membrane oxygenation can provide full cardiopulmonary support, has been increasingly used in the last two decades, and can be used to restore systemic end-organ hypoperfusion. However, a paucity of randomized controlled trials in combination with high complication and mortality rates suggest the need for more research to better define its efficacy, safety, and optimal patient selection. In this review, we provide an updated review on VA-ECMO, with an emphasis on its application in cardiogenic shock, including indications and contraindications, expected hemodynamic and echocardiographic findings, recommendations for weaning, complications, and outcomes. Furthermore, specific emphasis will be devoted to the two published randomized controlled trials recently presented in this setting.

## 1. Introduction

Cardiogenic shock (CS) is a life-threatening condition in which a low cardiac output results in insufficient end-organ perfusion and frequently requires hemodynamic support [1,2]. Hemodynamic criteria typically used to define CS in clinical trials have included systolic blood pressure < 90 mmHg for 30 min prior to the initiation of inotropes and vasopressors, cardiac index ≤ 2.2 L/min/m^2^, and elevated pulmonary capillary wedge pressure ≥15 mmHg [1,3]. End-organ hypoperfusion, including tissue ischemia, altered mental status, oliguria, elevated arterial lactic acid levels, and multiorgan failure, are fundamental features of CS and can also aid in establishing the diagnosis [2]. CS can occur in the setting of an acute cardiac event or due to decompensation of underlying chronic cardiomyopathy [4]. The leading cause, accounting for approximately 80% of cases, is acute myocardial infarction (AMI), and patients presenting with ST-segment elevation myocardial infarction are at greatest risk [5,6]. Other common causes include chronic heart failure, fulminant myocarditis, valvular heart disease, high-risk pulmonary embolism, and hemodynamically unstable arrhythmias, and the incidence of these etiologies is increasing [2,7]. CS accounts for an estimated 100,000 hospital admissions annually, and despite significant advances achieved in reperfusion therapy and mechanical circulatory support (MCS), CS remains associated with a high rate of complications and excessively high patient morbidity and mortality [4,8].

Extracorporeal membrane oxygenation has been increasingly used in the last two decades and has been shown to be an effective tool in the management of CS [2,9,10]. Venoarterial (VA) ECMO provides rapid, robust biventricular circulatory support and ventilatory support with impaired cardiac output [3,11]. It allows time for diagnostic and definitive therapeutic interventions and potential organ recovery, often serving as a bridge to recovery, bridge to further decision-making, or bridge-to-destination therapy [1,10,12]. Although the utilization and understanding of VA-ECMO is increasing and the Extracorporeal Life Support Organization (ELSO) has proposed several general recommendations, the scarcity of data from controlled trials for management, in combination with high complication and mortality rates, suggest ongoing challenges and continued room for improvement [4,10]. In this article, we provide an updated, comprehensive review on VA-ECMO and evaluate the current literature for its application in CS.

## 2. VA-ECMO

**Figure 1:**
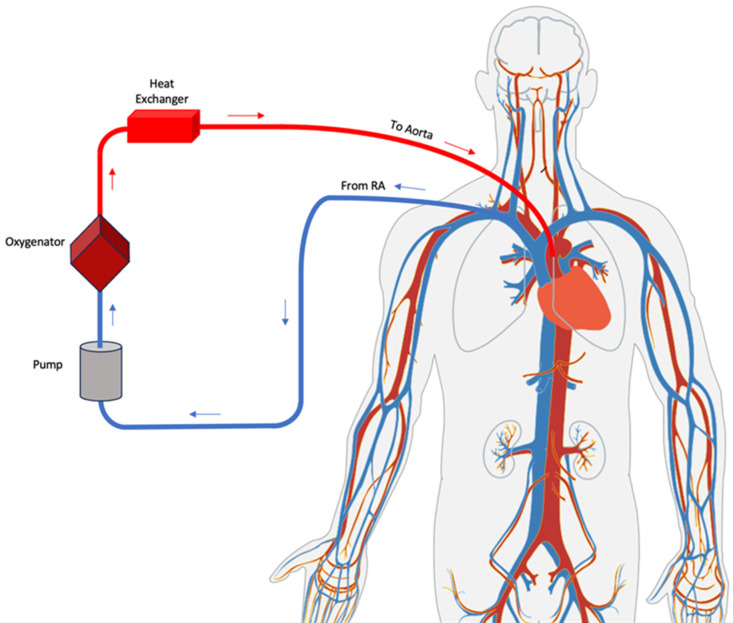
VA ECMO central configuration.

VA-ECMO, also commonly referred to as extracorporeal life support (ECLS), is a form of cardiopulmonary bypass that uses a centrifugal flow pump, membrane oxygenator, as well as venous inflow and arterial outflow cannulas [3,11]. Additional ports may also be added to the ECMO machinery to be used for ultrafiltration and hemodialysis [13]. In ECMO, deoxygenated blood drained from a central vein is passed through the membrane oxygenator, which is responsible for helping normalizing the pCO2, pO2, and pH, and is then pumped back into the systemic circulation via the centrifugal pump. Cardiac support can be up to 6–7 L of blood per minute [2,14]. The pump can be set to either partially or completely unload the heart by adjusting flow and left ventricular unloading configurations [15] (Figure 1).

**Figure 2:**
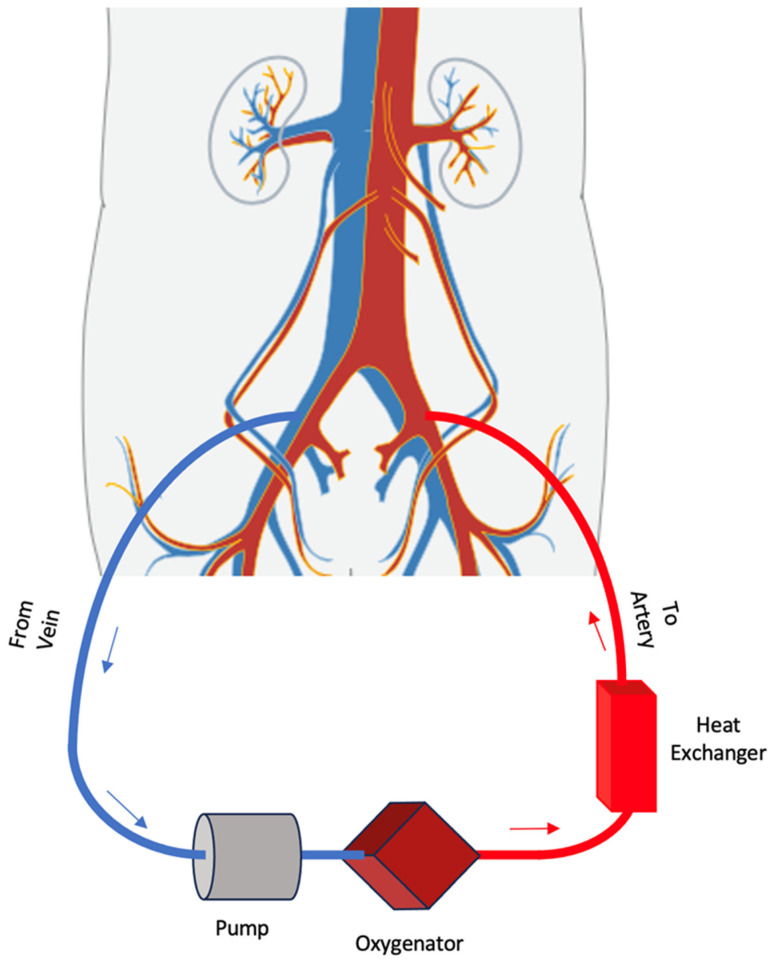
VA ECMO peripheral configuration.

Cannulation may occur centrally or peripherally [11,16]. Appropriate selection of cannula size is critical to reduce the risk of vascular injury and avoid negative inflow and high outflow pressures: 18–28 Fr venous and 15–19 Fr arterial cannulas are most commonly used [2]. In central cannulation, the venous inflow cannula is placed directly into the right atrium and arterial outflow directly into the ascending aorta, which allows for physiological anterograde circulation [16,17]. However, given the invasive nature of the procedure, central cannulation is performed in the operating room and most frequently occurs in patients who are unable to be weaned off cardiopulmonary bypass after cardiotomy [11,16]. Peripheral cannulation is typically performed with a percutaneous approach or surgical grafting of the peripheral vasculature [18]. In the femorofemoral configuration, the inflow and outflow sites are the femoral vein and artery, respectively, therefore resulting in perfusion in the retrograde direction [16]. Peripheral cannulation may also involve the arteries of the upper extremity—the axillary, subclavian, or carotid arteries—allowing for anterograde perfusion, improved cerebral perfusion, and increased patient mobility [11,16]. Additionally, unlike with central cannulation, peripheral cannulation via the femoral approach can be performed safely outside of the operating room, including in the catheterization laboratory, at the bedside in the ED or ICU, or even remotely “in the field” during patient stabilization and transfer [10,19,20] (Figure 2).

It is important to note that VA-ECMO can also be accomplished via a novel ambulatory approach. In ambulatory ECMO, cannulation in the groin is avoided and sufficient oxygen is provided to allow patients to stand, walk, and participate in active physiotherapy and thus help prevent deconditioning [21,22]. Although no studies have been completed to date to assess its safety and efficacy in patients with CS, there have been small studies showing safety and feasibility in carefully selected patients, and several case reports have shown success when used as a bridge to cardiac transplant [23,24]. Most of the current understanding and experience with ambulatory ECMO has been in patients in respiratory failure on venovenous (VV) ECMO awaiting lung transplant [21,25]. However, given that ambulatory ECMO is associated with minimized deconditioning, improved rates of return to independent functioning, decreased rates of delirium, and shorter ICU and hospital lengths of stay, it may become more commonplace in the treatment of patients with CS in the future [23,26].

Once ECMO is initiated, frequent monitoring of hemodynamics and assessment of arterial and venous blood gases, as well as gas samples from the VA-ECMO circuit, are essential to ensure that the cardiac output and oxygenation can promote myocardial recovery and help restore renal, hepatic, and pulmonary function, acid–base balance, coronary perfusion, and neurological status [4,10,27]. It is generally recommended that the flow target be in the range of 4–6 L/min, mean arterial pressure (MAP) target above 60 mmHg, arterial oxygen saturation target above 90%, the venous saturation target above 60%, although there is currently limited literature and lack of standardized guidelines regarding optimal titration and management [27,28,29,30,31]. Nonetheless, the utilization of VA-ECMO for CS has been rapidly increasing over the last two decades in the ELSO registry. With use in over 15,000 adult patients—an estimated increase of over 1000%—analysis of these cases may provide sufficient data to propose specific standardized guidelines for optimal patient management in the near future [11,32,33].

## 3. Indications and Contraindications of VA-ECMO

Patient selection is an important, though clinically challenging component of VA-ECMO use that aims to identify patients with the highest chance of recovery or candidacy for destination therapy [10,11,34]. ELSO has suggested guidelines for indications and contraindications; however, the decision to initiate VA-ECMO should be individualized [4,34]. Comorbidities, patient-specific risk factors, weaning strategies, and overall prognosis need to be considered, as patients with a low likelihood of recovery are unlikely to benefit from the invasive method and may be best managed with a conservative approach (Table 1) [1,4,10].

Indications for VA-ECMO include CS refractory to conventional medical and device-based therapy, most commonly in the setting of acute coronary syndrome, acute on chronic decompensated heart failure, fulminant myocarditis, and unsuccessful cardiopulmonary bypass weaning following cardiotomy [10,11,31]. Less commonly seen, but increasing in incidence is the use in CS due to pulmonary hypertension with subsequent cor pulmonale and due to PE with hemodynamic compromise [4]. VA-ECMO can also be used in cardiac arrest through extracorporeal cardiopulmonary resuscitation (ECPR), as well as in cases where temporary mechanical support is needed as a bridge prior to left ventricular assist device (LVAD) placement or cardiac transplant [2,4]. It has been reported that VA-ECMO can be an effective treatment for ventricular septal rupture and severe primary graft dysfunction after cardiac transplantation [35,36,37,38]. In addition, there have been case reports describing the use of VA-ECMO in the management of COVID-19-associated acute myocardial injury complicated by cardiogenic shock, though additional studies are needed in order to accurately assess its safety and in this subset of patients [39,40]. Lastly, the use of VA-ECMO in patients with sepsis remains controversial, though it may be appropriate in carefully selected patients with refractory septic shock [41].

Overall, ECMO should not be used as therapy in patients with cardiac disease that is unlikely to recover, as well as in patients with a poor life expectancy, typically less than one year, and in patients with preexisting conditions that have a very high mortality rate and that are incompatible with ECMO weaning and recovery, most notably severe neurological injury, disseminated malignancy, and irreversible multiorgan failure [2,17]. Additional absolute contraindications include unwitnessed or prolonged cardiac arrest as well as incompatible patient goals of care, such as “do not resuscitate” (DNR) orders [1,2]. VA-ECMO should also be avoided in patients with severe aortic insufficiency, as the increase in afterload puts the patients at risk of further hemodynamic compromise [10]. It may also be contraindicated in patients who cannot be anticoagulated, as therapeutic anticoagulation is currently standard practice with VA-ECMO [2,10]. Furthermore, for the femorofemoral approach specifically, the presence of a vena cava filter and severe aortoiliac disease are additional contraindications. Advanced age, cognitive impairment, medical comorbidities, poor compliance, and inadequate social support are additional relative contraindications that need to be considered [31,42]. Although age alone is not a contraindication to VA-ECMO, studies have consistently reported that advanced age is an independent predictor of in-hospital mortality [42,43]. To aid physicians in selecting appropriate patients and identifying those at risk of poor outcomes, several clinical indices—including the Survival After VA-ECMO (SAVE) score and the new simplified cardiac ECMO score—have been proposed to assess the likelihood of in-hospital mortality and predict recovery and hospital discharge [11,44,45]. Although the scores may prove to be beneficial, because they have been validated in people who have been put on VA-ECMO, their drawback is that they come with an inherent selection bias. In all cases, the risk factors, potential benefit, patient prognosis, comorbidities, and weaning approaches need to be considered in each individual patient prior to VA-ECMO initiation [4,17].

## 4. Hemodynamic Findings

Cardiogenic shock is the most severe form of LV failure, in which systolic or diastolic dysfunction leads to diminished cardiac output (CO), most often due to a decrease in contractility and a subsequent severe reduction in LVEF [11,46,47,48]. Reduced CO, low cardiac index, typically below 2.2 L/min/m^2^, and a profound fall in blood pressure lead to low systemic and coronary perfusion, triggering reflex-mediated increases in heart rate and systemic vascular resistance (SVR) [11,49]. In the classic paradigm of cardiogenic shock, the compensatory sympathetic stimulation contributes to worsening cardiac dysfunction, as the increase in heart rate and contractility increases myocardial oxygen demand, and systemic vasoconstriction and increase in SVR lead to an increase in the functional circulating blood volume, up to 50% of total blood volume, elevating the biventricular afterload and LV end diastolic pressure [46,50]. Volume overload is further exacerbated by an augmented preload, due to renal salt and fluid retention through activation of the renin–angiotensin–aldosterone system [50]. Ultimately, the resulting hypotension, tachycardia, and decreased coronary perfusion in the setting of increased myocardial oxygen demand exacerbate myocardial ischemia and dysfunction, further deteriorate myocardial contractility, and lead to a vicious cycle of declining CO, SV, and BP and increasing LV volume, resulting in progressive end-organ hypoperfusion, and if not resolved, eventual death [46,49,50].

The hemodynamic effects of a mixed shock state also need to be considered, as approximately 20% of patients admitted to the cardiac intensive care unit have this form of shock [51]. Typically, the mixed state is a combination of cardiogenic and distributive shock, which may occur in the setting of systemic inflammation or sepsis, and results in pathological vasodilation in the setting of reduced cardiac output. Acute cardiac injury can trigger capillary leakage and the release of inflammatory mediators, which can lead to systemic vasodilation, decreased SVR, and exacerbate hypotension [5]. It is estimated that almost a fifth of patients presenting with an AMI have a vasodilatory shock component due to myonecrosis induced inflammatory changes [5]. The resulting tissue underperfusion leads to the formation of lactic acid, additionally contributing to cardiac dysfunction [11,47]. Nonetheless, despite certain differences in the hemodynamics, in both cases of cardiogenic and mixed shock, low cardiac output and decreased coronary perfusion lead to progressive cardiac dysfunction, and if uncorrected, end in death.

The specific hemodynamic effects of VA-ECMO on the heart and cardiovascular system in CS are still being analyzed. It is known that VA-ECMO reduces central venous pressure while increasing MAP and the arteriovenous pressure gradient, thereby increasing systemic perfusion [2]. One theory suggests that VA-ECMO reduces right ventricular (RV) preload, RV blood flow into the pulmonary artery, and peripheral venous congestion, which results in a decrease in LV end diastolic volume and pressure and promotes hemodynamic stabilization [2,6,11,52]. However, another proposes that VA-ECMO increases cardiac afterload, which subsequently results in a rise in LVEDP, left atrial pressure, and pulmonary capillary wedge pressure, contributing to worsening of LV function and pulmonary edema [4,6,14,53]. It has been estimated that up to 30% of patients placed on VA-ECMO will exhibit pulmonary edema [17,53,54,55]. Furthermore, especially in patients with no native cardiac ejection or those with severe LV dysfunction placed on high flow rates, the significant increase in afterload may result in insufficient opening of the aortic valve (AV), LV blood accumulation, and LV distention, further contributing to worsening of pulmonary edema [11,18,52]. Additionally, the increased afterload and LV distention in the setting of elevated LV filling pressures decreases the transcoronary perfusion gradient, leading to impaired myocardial perfusion and worsening dysfunction [11].

The major disadvantage with peripheral VA-ECMO is that it lacks complete LV “unloading” capabilities—it does not lessen the work of the LV—therefore, optimization of preload, afterload, and contractility may be needed to maintain forward flow through the LV and prevent pulmonary edema and decreased LV function [6,17,18]. When medical therapy with diuretics and inotropes are insufficient, mechanical means, or LV “venting” strategies, are often used [3,18].

According to the ELSO, of the 12,734 adult patients who received VA-ECMO between 2010 and 2019, 3399 patients required mechanical unloading, 82.9% with the intra-aortic balloon pump (IABP) and 17.1% with transvalvular percutaneous ventricular assist device (pVAD), such as the Impella [56]. The IABP, a percutaneous device placed into the descending aorta, can be an effective tool for LV unloading due to its ability to improve the myocardial oxygen supply-to-demand ratio. It increases the coronary and myocardial perfusion while decreasing the left ventricular afterload during systole and thus reduces the myocardial work [57,58]. The most recent systematic meta-analysis by Zeng et al. examining nine manuscripts and over 2500 patients found a significant in-hospital survival benefit in CS patients on VA-ECMO in combination with IABP compared with VA-ECMO alone, with comparable rates of adverse bleeding and infection [59]. The pVAD, a catheter-based miniaturized ventricular assist device that is placed across the aortic valve and into the LV, works to unload the ventricle by maintaining a systemic circulation via actively pumping blood from the LV into the ascending aorta [60,61]. Fiorelli et al. conducted a meta-analysis examining outcomes in VA-ECMO in combination with the Impella (ECPELLA) vs. VA-ECMO alone in 972 CS patient across five studies. They found that LV unloading with ECPELLA was associated with lower mortality rates—56.1% compared to 63.7% in the control [54,57,58,62].

Less commonly utilized means of unloading the LV include atrial septostomy, left atrial or pulmonary artery VA-ECMO, and an LV direct surgical vent [3,18,62]. Surgical or percutaneous balloon atrial septostomy creates a left-to-right shunt, which provides an immediate reduction in preload and afterload and decreases ventricular workload [63]. A multicenter registry of 223 patients who underwent atrial septostomy for VA-ECMO unloading showed that septostomy was associated with significant complications, including arrhythmia and tamponade, and had an overall hospital mortality rate of 46% [64]. Although there are no randomized or systematic trials examining efficacy and mortality of LA and pulmonary artery VA-ECMO, small, single-center cases have reported they are effective methods for LV unloading and can be utilized for successful weaning from VA-ECMO [65,66,67]. Partial ECMO flow is an additional venting strategy to prevent LV distention, while allowing ejection from the LV [68,69,70].

Although multiple studies have reported lower mortality and higher rates of weaning in adult patients with CS treated with VA-ECMO in combination with LV mechanical unloading, there are currently no randomized trials comparing the various venting strategies [71,72]. Early detection of LV distention and intervention is important. Diagnostic modalities to evaluate for LV distention include X-ray, arterial line wave form, pulmonary artery catheter, and monitoring of clinical symptoms, including bloody secretions.

A further hemodynamic concern is the development of “north–south syndrome,” also known as “harlequin syndrome,” which has been reported to occur in up to 8.8% of patients on VA-ECMO [73,74]. This rare phenomenon occurs in patients with femoral artery cannulation, where well-oxygenated blood from the VA-ECMO circuit is returned in a retrograde fashion up the aorta and mixes with poorly oxygenated blood from the native circulation, often in patients with pulmonary compromise in which gas exchange is severely impaired [74,75]. As the cardiac function improves or supplemental left-sided mechanical support devices for ventricular unloading are introduced, the outflow from the native LV can overcome the retrograde flow from the circuit and lead to selective hypoxia, with poorly oxygenated blood, often below 90% saturation, perfusing the brain, coronary arteries, and upper extremities [10]. In this condition, switching to central cannulation or peripheral cannulation from the upper extremities is an option. Though there are currently no standardized criteria to diagnose harlequin syndrome, detection of an arterial oxygen saturation gradient higher than 15% between right and left radial arteries is suggestive of the syndrome, and opting to switch from femoral cannulation to central or upper extremity cannulation may be beneficial [76].

The hemodynamic benefits of VA-ECMO are irrespective of intrinsic LV function and have an advantage over IABP and Impella devices alone by functioning regardless of RV function due to bypassing the pulmonary circuit for oxygenation [14]. Thus, unlike IABP or Impella support alone, VA-ECMO may be used in refractory biventricular failure [2]. Ultimately, despite recognized benefits of the use of VA-ECMO in patients with cardiogenic shock, the hemodynamic responses of this group of patients are variable and complex, and critical gaps remain in our understanding.

## 5. Echocardiographic Findings

Echocardiography plays an important role in VA-ECMO management. Although no specific guidelines currently exist, assessment with the imaging modality provides anatomical and diagnostic information that can aid in patient selection, facilitates safe cannulation and weaning, and serves as a standardized tool to monitor patients and evaluate for complications [52,77]. Comprehensive echocardiographic evaluation, either by transthoracic echocardiography [78] or transesophageal echocardiography [78], should be completed in all VA-ECMO candidates, though the assessment may need to be omitted in patients with hemodynamic instability in urgent need of MCS cannulation [53]. Echocardiography is able to establish baseline anatomy, including LV size and wall thickness, and objective measurements of systolic and diastolic function, including LVEF, which can serve as a reference in assessing myocardial recovery. Furthermore, TTE/TEE provides a thorough assessment of valvular morphology and competence and can recognize underlying structural defects or the presence of mechanical valves [52,53]. Insufficiency of the aortic and mitral valves especially need to be identified and quantified, as initiation of VA-ECMO may worsen the degree of preexisting regurgitation due to the significant increase in afterload [52]. Lastly, an echocardiograph may be able to determine the precise etiology of CS or identify any potential contraindications to VA-ECMO initiation [53].

Once it is determined that VA-ECMO is indicated, cannulation can be performed under fluoroscopy, TTE, or TEE guidance to allow for direct visualization of the guidewire, ensure appropriate cannula placement, and promptly identify complications during insertion and positioning, including life-threatening pericardial effusion, aortic dissection, and stroke or other embolic event [52,53]. These modalities may not be available during ECPR due to ongoing CPR, which makes cannulation more challenging. After VA-ECMO is initiated, serial evaluations with daily TEE are the most reliable method to ensure sufficient emptying of the ventricles and monitor left ventricular function, distension, and degree of unloading [17,52]. With increasing VA-ECMO flow rates, the aortic pressure increases, which leads to increased LV volume and distension. On TEE, this is characterized by a dilated and impaired LV, significant mitral regurgitation during systole and diastole, and in severe cases, failure of aortic valve (AV) opening [14,52,53]. A closed AV increases the risk of thrombus formation due to blood stasis, which on TEE is depicted as an intracavitary spontaneous echo contrast [53]. Intracardiac thrombi account for approximately 5% of all VA ECMO complications and may be intracavitary, most often found in the left-sided heart chambers, or along the aortic root in cases where the LV is not vented or has significantly diminished LV ejection [10,18,79]. Though they have potential to embolize and increase the risk of cerebral, renal, and mesenteric ischemia, which can significantly contribute to increased mortality, echocardiography has been reported to be an effective tool in prevention and diagnosis [53,80]. Monitoring with TEE can also aid in detecting ECMO dysfunction and complications, including cannula displacement, cardiac tamponade, vascular obstruction, or cannula-associated thrombi, including a PE [77]. Lastly, echocardiography can facilitate clinical decision-making regarding circulatory support weaning, as it is able to track LV function from baseline through various VA-ECMO flow rates, and thus helps in assessing cardiac recovery [77]. Improvement in LVEF, absence of LV dilation, increased AV opening, and left ventricular outflow tract velocity time integral—a measure of cardiac systolic function and cardiac output—above 10 cm on TEE are all indicators of LVEF improvement [52,81].

## 6. Complications

VA-ECMO is a promising form of MCS in patients with CS; however, utilization, must be carefully weighed against potential complications, many of which can significantly increase the risk of morbidity and mortality [4,10].

Because therapeutic anticoagulation is standard practice with VA-ECMO, bleeding is the most commonly reported adverse event, reportedly occurring in 30–79% of patients with VA-ECMO use [4,29,82,83,84]. Bleeding is frequently reported at cannulation sites; however, VA-ECMO may also lead to systemic hemorrhage, most commonly in the upper and lower gastrointestinal tract, thorax, and pericardium [2,10]. The ECMO circuit can also contribute to hemolysis and thrombocytopenia, increases the risk of disseminated intravascular coagulation and heparin-induced thrombocytopenia, and given the disproportionately high shear stress it causes within the cardiovascular system, can result in acquired von Willebrand factor deficiency. An increased number of units transfused is associated with a higher mortality rate [10].

Risk of hemorrhagic complications must be balanced with risk of thrombosis, as thrombotic complications are also regularly encountered, occurring in up to 22% of patients [4,29,83,85,86,87]. A prothrombotic inflammatory environment can result from blood exposure to the VA-ECMO artificial surfaces as well as intraventricular or aortic root blood stagnation, and may result in both thromboembolic events in the patient and VA-ECMO pump malfunction [11]. There is currently no clear consensus on anticoagulation strategy and management differs significantly between patients, though current ELSO guidelines for anticoagulation during ECMO recommend an initial heparin infusion rate of 7.5–20.0 units/kg/h [88,89]. Furthermore, the conventional recommendation, based largely on expert opinion, is regular monitoring of coagulation studies and using unfractionated heparin to target an activated clotting time of 180 to 220 s, a partial thromboplastin time (aPPT) target in the 60–80 range, and anti-Xa level in the 0.3–0.7 IU/mL range [2,4,10,11,89,90,91]. Although anticoagulation has been thought to be standard practice, a recent report demonstrated the safety and efficacy of VA-ECMO support without anticoagulation. Patients receiving no anticoagulation had comparable mortality rates and lower overall complication rates—including bleeding—compared to their anticoagulated counterparts [68,92]. Overall, preventing bleeding and thrombosis is challenging as it requires finding the optimal balance between anticoagulation and hemostasis. Thoughtful review of patient medications and medical history, meticulous surgical or cannulation technique, close monitoring of patient clinical presentation, medication, and lab work, as well as an interdisciplinary approach, including an expert in hemostasis, can aid in preventing hemorrhagic or coagulant complications [68,93,94].

Limb ischemia is also a known complication of VA-ECMO, and its associated mortality rate is reported to be as high as 60% [10,11,34]. Percutaneous or surgical placement of a distal perfusion cannula is performed to provide antegrade femoral blood flow to the cannulated leg and lower the risk of ischemic limb injury, though it is still reported to occur in 13–35% of patients with peripheral VA-ECMO [4,11,17,29]. Some centers advocate aggressive placement of a distal perfusion catheter, while other centers use a selective approach with careful monitoring of limb ischemia by using either a somatic oximetry sensor or near-infrared spectroscopy (NIRS) [54,95,96,97,98,99]. It is also important to keep in mind that limb ischemia can occur as a result of severe cardiogenic shock, vasoconstriction, or hypothermia. To rule out acute limb ischemia, physical examination, Doppler pulse check, and ultrasound are paramount. Furthermore, the frequency of compartment syndrome and need for fasciotomy and lower extremity amputation, respectively, is reported to be 7.3–14.5% and 2.3–9.3% [2]. Prevention of vascular complications related to VA-ECMO is crucial, as these adverse events are significantly associated with survival [98].

Acute kidney injury (AKI) is another frequently encountered complication. The incidence is estimated to be between 43–85% and typically occurs within 48 h of VA-ECMO cannulation [34,100,101]. The pathophysiology of ECMO-associated AKI is complex and multifactorial, though the hemodynamic changes that occur with vasopressors and inotrope use, as well as with ECMO cannulation, and the subsequent changes in renal blood flow resulting in ischemia and reperfusion, are thought to play a major role [100,101,102]. It has been suggested that severity of kidney dysfunction at ECMO initiation is a strong predictor of long-term survival, as single-center studies have shown that patients with AKI who require renal replacement therapy had an 80% mortality rate compared with a mortality rate of 20% in non-AKI patients [11,84,100,102]. However, it is important to note that because AKI and subsequent renal failure is often one of the early signs of multiorgan failure and death, it is unclear whether AKI and renal failure directly increase the risk of mortality or whether they simply correspond to the severity of the critical illness [34,54,101,103]. Continuous renal replacement therapy (CRRT) can be undertaken through an integrated approach within the VA-ECMO circuit or via a parallel system with separate VA-ECMO and CRRT circuits [54,101,103]. However, although studies have reported that CRRT is safe and feasible in patients with VA-ECMO, data regarding renal recovery and overall outcomes continue to be limited [101].

Other common complications of VA-ECMO include infections, most commonly accessed site, bloodstream, and lower respiratory and urinary tract infections, which are reported to occur in up to 20% of patients and can be prevented by utilizing strict aseptic technique. Additionally, patients can experience neurological complications, such as acute ischemic stroke, intracerebral hemorrhage, seizure, and anoxic brain injury; These can be mitigated with an improved understanding of potential neurological complications that can be expected with VA-ECMO that can lead to morbidity and mortality, as well as with regular and multimodal monitoring, in order to arrive at an early diagnosis and initiate timely treatment [10,68,104,105,106].

## 7. Weaning

VA-ECMO serves as a window during which the decision to proceed with durable LVAD or cardiac transplantation is performed or reversible causes of cardiac failure can be treated [31]. Weaning from support is considered once there are signs of myocardial recovery: the initial condition requiring VA-ECMO has resolved or improved, and vasoactive medications are significantly reduced or no longer needed [2,53]. The weaning process and evaluation of improvement in cardiac function can be supported with serial echocardiography, both TTE and hemodynamic TEE, and invasive hemodynamic monitoring [10,52,107,108]. Nonetheless, determining the optimal weaning strategy is challenging, as there are currently no randomized clinical trials and physicians heavily rely on expert opinion and their own clinical decision-making in order to balance the risks of premature weaning, including cardiac compromise from high-dose inotropes, hemodynamic instability, or need for emergent recannulation, with the risks of delayed weaning, most notably prolonging exposure to VA-ECMO and its associated complications and high risk of morbidity and mortality [52,107,109]. The most recent guidelines, proposed in 2022 by the American Heart Association (AHA), recommend daily assessment of cardiac function, with the goal of withdrawing VA-ECMO as soon as patients show improvement in the underlying cause of their CS, are intravascularly euvolemic, and are hemodynamically stable with minimal intravenous support. A stepwise decrease in support flow is recommended, typically a reduction in increments of 0.5 to 1 L/min until the level of 1.5 to 2.0 L/min, at which point decannulation can occur. The standard frequency of the stepwise flow reduction is every 2 to 4 h; however, weaning may occur more rapidly, every 5 to 15 min, in a small subset of patients, including patients with CS due to AMI following revascularization and LVEF recovery and patients with CS due to valvular lesions that have been corrected [78].

Several other weaning algorithms have been proposed, though the basis of each strategy includes weaning trials, in which the performance of the ventricles and patient hemodynamic response is assessed throughout an incremental decrease in support in order to determine whether VA-ECMO can safely be terminated [2,107]. There are several considerations that need to be addressed prior to the initiation of the weaning process. Initially, the prospective recovery of the underlying cause of CS needs to be evaluated. Next, clinical, hemodynamic, and echocardiographic data should be consistent with myocardial recovery significant enough to ensure sufficient end-organ perfusion and meet the metabolic demands of the body [10]. Any metabolic disturbances or end-organ dysfunction should be recovered or supported by other means [10,54]. Furthermore, pulmonary function should not be severely impaired; pulmonary oxygenation with a PaO_2_/FiO_2_ greater than 200 on 0.21 FiO_2_ is recommended, and transition to VV-ECMO should be considered in patients with PaO_2_/FiO_2_ less than 100 [52,54,109]. Lastly, the patient should have recovered a pulsatile arterial waveform for at least 24 h and be hemodynamically stable [109,110]. The baseline mean arterial pressure (MAP) should be greater than 60 mmHg in the absence of or with low doses of catecholamines and vasopressors, though data suggest that better outcomes are linked with lower levels of pharmacological hemodynamic support at time of weaning [10,110]. It is common to use another form of temporary MCS when VA-ECMO weaning is attempted, for example, percutaneous devices such as IABP or the Impella, and many patients are weaned from VA-ECMO with these devices still in place.

Once these criteria are met and patients are deemed ready for weaning, an algorithmic approach is recommended for incrementally decreasing the bedside VA-ECMO blood flow rate, exposing the patient to an increased RV preload and decreased LV afterload [15]. In patients with CS, VA-ECMO flow support is typically run at approximately 3 to 4 L/min, although higher rates may be necessary [11]. With weaning, the flow rate is gradually decreased to a fraction of its baseline value, and then to a minimum of 1 to 1.5 L/min [11,17,53]. Throughout this process, cardiac function is assessed using hemodynamic and echocardiographic data to determine whether the degree of myocardial recovery will allow for complete removal of VA-ECMO. LVEF above 30%, maintenance of mean arterial pressure, and left ventricular ejection above the ECMO flow rate are all indicative of tolerating VA-ECMO weaning [17]. In these patients, a complete wean is scheduled in an operating room to allow for controlled decannulation or expedited recannulation and MCS initiation if removal of VA-ECMO is not tolerated [10].

The precise criteria to define successful weaning have not been established, though VA-ECMO device removal with no further necessity for MCS in the following 30 days for refractory CS is generally accepted [109]. Successful weaning from VA-ECMO is multifaceted and difficult to predict [109,111]. The reported rates of successful weaning in the literature range from 31% to 76% [2,112]. For patients who cannot be weaned off ECMO, LVAD, cardiac transplantation, or end-of-life care need to be considered [10,17].

## 8. ECMO Outcomes in Cardiogenic Shock

Based on retrospective and observational studies, morbidity and mortality remain exceptionally high in patients requiring VA-ECMO, and outcomes are largely dependent on the underlying indication, patient comorbidities, severity of organ dysfunction at initiation, and complications or adverse events during MCS [20,109]. The reported overall survival to hospital discharge has been reported to be between 29% and 63.1% [11,83,84,113,114,115]. A systematic review of 24 studies and nearly 2000 patients found the survival rate at discharge to be 30.8% [116]. It should be noted that between 20% and 65% of patients successfully weaned from VA-ECMO support do not ultimately survive to hospital discharge, mainly due to concomitant neurological injuries and multisystem organ failure, and the overall in-hospital mortality rate in patients requiring VA-ECMO for CS is estimated to be as high as 60% [8,11,109,112,114,117]. The one-year survival rate following successful VA-ECMO treatment was reported to be 73.7% in patients with ischemic heart disease and 75% in their non-ischemic heart disease counterparts [12]. Various predictors of in-hospital mortality have been described, and several variables have been linked with an increased risk of patient mortality [34]. Advanced age, diabetes, obesity, poor LVEF, renal insufficiency, elevated lactate, metabolic acidosis, elevated CK-MB at admission, greater use of inotropes and vasopressors, and ECMO insertion outside the operating room have all been linked with poorer outcomes [20,68,114,118,119].

To date, there have been two randomized controlled trials (RCT) examining VA-ECMO use in patients in CS: the Extracorporeal Membrane Oxygenation in the Therapy of Cardiogenic Shock (ECMO-CS trial) and the Venoarterial Extracorporeal Membrane Oxygenation or Standard Care in Patients with Cardiogenic Shock Complicating Acute Myocardial Infarction (EURO SHOCK trial).

The ECMO-CS trial examined the effectiveness of VA-ECMO in patients with rapidly deteriorating or severe cardiogenic shock. Specifically, the study compared outcomes in patients randomly assigned to immediately start VA-ECMO with those who were assigned to start with conservative management (CM) with delayed VA-ECMO initiation in the case of hemodynamic worsening. Of the patients in the CM group, 39% were ultimately started on VA-ECMO, an average of 1.9 days following randomization. The incidence of adverse events (61.3% in VA-ECMO; 61.0% in CM), as well as all-cause mortality at 30 days (50.0% in VA-ECMO, 47.5% in CM), was similar between the two groups, suggesting that immediate initiation of VA-ECMO in rapidly progressing or severe cardiogenic shock did not improve outcomes [120].

The EUROSHOCK trial was a prospective, multicenter RCT that examined outcomes of VA-ECMO vs. standard therapy in 35 patients with CS 30 min following percutaneous coronary intervention, of which 17 were randomized to the VA-ECMO group and 18 to standard therapy. The all-cause mortality at 30 days was 43.8% in the VA-ECMO group compared with 61.1% in the control, and at one year 51.8% and 81.5%, respectively. The rate of adverse events was found to be significantly higher in the VA-ECMO group: 21.4% of patients experienced vascular complications and 35.7% had bleeding complications, compared with 0% and 5.6% in the standard therapy group. Although the survival outcomes suggest a benefit in using VA-ECMO, given the limited sample size and risk of complications, definitive recommendations cannot be made based on this trial [121].

It is also important to note that the addition of VA-ECMO to CPR for cardiac arrest is reported to significantly improve patient outcomes [122,123]. Although RCTs are lacking, especially in the setting of CS, observational studies report an overall in hospital and out of hospital survival rate between 15% and 50% with ECPR compared with 10–20% in conventional CPR [122,124,125]. Chen et al. reported 34.1% overall survival to hospital discharge in patients with cardiac arrest undergoing ECPR [122,126]. Favorable neurological outcomes have also been reported with ECPR [125]. In an RCT by Belohlavek et al. examining 256 participants, it was found that 31.5% of patients receiving ECPR survived to 180 days with good neurologic outcomes compared with 22.0% in the standard CPR group [127]. Shin et al. reported a higher 2-year survival with minimal functional deficits in ECPR compared to traditional CPR in a study of 321 patients [122,128]. Lastly, a recent study by Tonna et al. examining over 1075 patients from over 200 centers identified six variables associated with in-hospital mortality—age, time of day, presenting rhythm, history of renal insufficiency, patient type, and cardiac arrest duration—and developed the RESCUE-IHCA score for bedside mortality prediction, which may be useful in identifying good candidates for VA-ECMO and ECPR [129].

In sum, despite the significant risk of mortality and potentially fatal complications, VA-ECMO offers a substantial chance of survival for patients in cardiogenic shock with an otherwise particularly poor prognosis [118]. Many questions remain regarding best utilization practices, though experts have traditionally agreed that prompt recognition of clinical deterioration and initiation of VA-ECMO in appropriate candidates allows for the greatest chance of survival and positive outcomes [54,130].

## 9. Conclusions

VA-ECMO provides rapid, complete biventricular circulatory support in addition to simultaneous gas exchange to allow time for diagnostic and therapeutic interventions and potential organ recovery, often serving as a bridge to recovery, bridge to further decision-making, or bridge to cardiac transplantation, and offers a chance of survival for patients in cardiogenic shock refractory to conventional medical and device-based therapy with an otherwise poor prognosis. Despite recognized benefits of the use of VA-ECMO, utilization must be carefully weighed against potential complications and patient selection is an important component of VA-ECMO use that aids in optimizing patient outcomes, while avoiding medical futility. Outcomes following VA-ECMO are largely dependent on the underlying indication, patient comorbidities, severity of organ dysfunction at initiation, and complications or adverse events during MCS. Significant advances have been achieved in our understanding of VA-ECMO; however, rigorous investigation with prospective, randomized controlled trials are needed in order to establish standardized evidence-based guidelines for optimal management of patients with cardiogenic shock requiring VA-ECMO.

## Figures and Tables

**Table 1:** Common indications, contraindications, and considerations for patient selection in VA-ECMO.

Patient Selection	
Patient-Specific Risk Factors, Potential Benefit, Patient Prognosis, Comorbidities, and Weaning Approaches Need to Be Considered in Each Individual Patient Prior to VA-ECMO Initiation	
Indications	Contraindications
*Cardiogenic Shock Refractory to Conventional Medical and Device-Based Therapy*	*Patients with an Overall Poor Prognosis and at High Risk of Morbidity and Mortality*
Acute or chronic decompensated HF Fulminant myocarditis Unsuccessful post-cardiotomy cardiopulmonary bypass weaning Pulmonary hypertension with subsequent cor pulmonale PE with hemodynamic compromise Cardiac arrest Ventricular septal rupture and severe primary graft dysfunction after cardiac transplantation COVID-19-associated acute myocardial injury Additional cases where temporary mechanical support is needed to bridge to LVAD or cardiac transplant	***Absolute*** Cardiac disease that is unlikely to recover Poor life expectancy, typically less than one year Preexisting conditions that have an expected mortality rate greater than 95% Preexisting conditions that are incompatible with ECMO weaning and recovery Neurological injury Disseminated malignancy Irreversible multiorgan failure Unwitnessed or prolonged cardiac arrest Incompatible patient goals of care, including DNR/DNI orders Significant aortic insufficiency Contraindication to anticoagulation ***Relative*** Advanced age Cognitive impairment Medical comorbidities Poor compliance Inadequate social support

## References

[1] Brugts J.J., Caliskan K. (2014). Short-term mechanical circulatory support by veno-arterial extracorporeal membrane oxygenation in the management of cardiogenic shock and end-stage heart failure. Expert Rev. Cardiovasc. Ther..

[2] Tsangaris A., Alexy T., Kalra R., Kosmopoulos M., Elliott A., Bartos J.A., Yannopoulos D. (2021). Overview of Veno-Arterial Extracorporeal Membrane Oxygenation (VA-ECMO) Support for the Management of Cardiogenic Shock. Front. Cardiovasc. Med..

[3] Telukuntla K.S., Estep J.D. (2020). Acute Mechanical Circulatory Support for Cardiogenic Shock. Methodist Debakey Cardiovasc. J..

[4] Mehta H., Eisen H.J., Cleveland J.C. (2015). Indications and Complications for VA-ECMO for Cardiac Failure. https://www.acc.org/latest-in-cardiology/articles/2015/07/14/09/27/indications-and-complications-for-va-ecmo-for-cardiac-failure.

[5] Van Diepen S., Katz J.N., Albert N.M., Henry T.D., Jacobs A.K., Kapur N.K., Kilic A., Menon V., Ohman E.M., Sweitzer N.K. (2017). Contemporary Management of Cardiogenic Shock: A Scientific Statement From the American Heart Association. Circulation.

[6] Vora N., Chaudhary R., Upadhyay H.V., Konat A., Zalavadia P., Padaniya A., Patel P., Patel N., Prajjwal P., Sharma K. (2023). Mechanical Assist Device-Assisted Percutaneous Coronary Intervention: The Use of Impella Versus Extracorporeal Membrane Oxygenation as an Emerging Frontier in Revascularization in Cardiogenic Shock. Cureus.

[7] Randhawa V.K., Sinha S.S., Hernandez-Montfort J. (2023). An Evolving Roadmap for Cardiogenic Shock Requiring Temporary Mechanical Circulatory Support. JACC Asia.

[8] Koerner M.M., Harper M.D., Gordon C.K., Horstmanshof D., Long J.W., Sasevich M.J., Neel J.D., El Banayosy A. (2019). Adult cardiac veno-arterial extracorporeal life support (VA-ECMO): Prevention and management of acute complications. Ann. Cardiothorac. Surg..

[9] Ivanov B., Krasivskyi I., Gerfer S., Sabashnikov A., Doss M., Holzhey D., Eghbalzadeh K., Rustenbach C., Kuhn E., Rahmanian P.B. (2022). Impact of Initial Operative Urgency on Short-Term Outcomes in Patients Treated with ECMO Due to Postcardiotomy Cardiogenic Shock. Life.

[10] Keebler M.E., Haddad E.V., Choi C.W., McGrane S., Zalawadiya S., Schlendorf K.H., Brinkley D.M., Danter M.R., Wigger M., Menachem J.N. (2018). Venoarterial Extracorporeal Membrane Oxygenation in Cardiogenic Shock. JACC Heart Fail..

[11] Rao P., Khalpey Z., Smith R., Burkhoff D., Kociol R.D. (2018). Venoarterial Extracorporeal Membrane Oxygenation for Cardiogenic Shock and Cardiac Arrest. Circ. Heart Fail..

[12] Ghodsizad A., Singbartl K., Loebe M., Zeriouh M., Ruhparwar A., Grant A., El-Banayosy A., Koerner M.M. (2017). Extracorporeal Membrane Oxygenation (ECMO): An Option for Cardiac Reccovery from Advanced Cardiogenic Shock. Heart. Surg. Forum.

[13] Medical Advisory Secretariat (2010). Extracorporeal lung support technologies—Bridge to recovery and bridge to lung transplantation in adult patients: An evidence-based analysis. Ont. Health Technol. Assess. Ser..

[14] Burkhoff D., Sayer G., Doshi D., Uriel N. (2015). Hemodynamics of Mechanical Circulatory Support. J. Am. Coll. Cardiol..

[15] Aissaoui N., Guerot E., Combes A., Delouche A., Chastre J., Leprince P., Leger P., Diehl J.L., Fagon J.Y., Diebold B. (2012). Two-dimensional strain rate and Doppler tissue myocardial velocities: Analysis by echocardiography of hemodynamic and functional changes of the failed left ventricle during different degrees of extracorporeal life support. J. Am. Soc. Echocardiogr..

[16] Shen J., Tse J.R., Chan F., Fleischmann D. (2022). CT Angiography of Venoarterial Extracorporeal Membrane Oxygenation. Radiographics.

[17] Ghodsizad A., Koerner M.M., Brehm C.E., El-Banayosy A. (2014). The role of extracorporeal membrane oxygenation circulatory support in the ‘crash and burn’ patient: From implantation to weaning. Curr. Opin. Cardiol..

[18] Piechura L.M., Coppolino A., Mody G.N., Rinewalt D.E., Keshk M., Ogawa M., Seethala R., Bohula E.A., Morrow D.A., Singh S.K. (2020). Left ventricle unloading strategies in ECMO: A single-center experience. J. Card. Surg..

[19] Ali J.M., Vuylsteke A., Fowles J.A., Pettit S., Salaunkey K., Bhagra S., Lewis C., Parameshwar J., Kydd A., Patvardhan C. (2020). Transfer of Patients With Cardiogenic Shock Using Veno-Arterial Extracorporeal Membrane Oxygenation. J. Cardiothorac. Vasc. Anesth..

[20] Pitsis A.A., Visouli A.N. (2011). Mechanical assistance of the circulation during cardiogenic shock. Curr. Opin. Crit. Care.

[21] Lindholm J.A. (2018). Ambulatory veno-venous extracorporeal membrane oxygenation. J. Thorac. Dis..

[22] Rao P., Alouidor B., Smith R., Khalpey Z. (2018). Ambulatory central VA-ECMO with biventricular decompression for acute cardiogenic shock. Catheter. Cardiovasc. Interv..

[23] Burkhart H.M., Thompson J.L., Mascio C.E. (2018). Ambulatory extracorporeal membrane oxygenation as a bridge to cardiac transplant: A step in the right direction?. J. Thorac. Cardiovasc. Surg..

[24] Hess N.R., Hickey G.W., Murray H.N., Fowler J.A., Kaczorowski D.J. (2022). Ambulatory simultaneous venoarterial extracorporeal membrane oxygenation and temporary percutaneous left ventricular assist device bridge to heart transplantation. JTCVS Tech..

[25] Garcia J.P., Kon Z.N., Evans C., Wu Z., Iacono A.T., McCormick B., Griffith B.P. (2011). Ambulatory veno-venous extracorporeal membrane oxygenation: Innovation and pitfalls. J. Thorac. Cardiovasc. Surg..

[26] Pasrija C., Mackowick K.M., Raithel M., Tran D., Boulos F.M., Deatrick K.B., Mazzeffi M.A., Rector R., Pham S.M., Griffith B.P. (2019). Ambulation With Femoral Arterial Cannulation Can Be Safely Performed on Venoarterial Extracorporeal Membrane Oxygenation. Ann. Thorac. Surg..

[27] Richardson A.S.C., Tonna J.E., Nanjayya V., Nixon P., Abrams D.C., Raman L., Bernard S., Finney S.J., Grunau B., Youngquist S.T. (2021). Extracorporeal Cardiopulmonary Resuscitation in Adults. Interim Guideline Consensus Statement From the Extracorporeal Life Support Organization. ASAIO J..

[28] Keller S.P. (2019). Management of Peripheral Venoarterial Extracorporeal Membrane Oxygenation in Cardiogenic Shock. Crit. Care Med..

[29] Lafc G., Budak A.B., Yener A.U., Cicek O.F. (2014). Use of extracorporeal membrane oxygenation in adults. Heart Lung Circ..

[30] Winiszewski H., Guinot P.-G., Schmidt M., Besch G., Piton G., Perrotti A., Lorusso R., Kimmoun A., Capellier G. (2022). Optimizing PO_2_ during peripheral veno-arterial ECMO: A narrative review. Crit. Care.

[31] Tariq S., Gass A. (2016). Use of Extracorporeal Membrane Oxygenation in Refractory Cardiogenic Shock. Cardiol. Rev..

[32] Guglin M., Zucker M.J., Bazan V.M., Bozkurt B., El Banayosy A., Estep J.D., Gurley J., Nelson K., Malyala R., Panjrath G.S. (2019). Venoarterial ECMO for Adults: JACC Scientific Expert Panel. J. Am. Coll. Cardiol..

[33] Mastoris I., Tonna J.E., Hu J., Sauer A.J., Haglund N.A., Rycus P., Wang Y., Wallisch W.J., Abicht T.O., Danter M.R. (2022). Use of Extracorporeal Membrane Oxygenation as Bridge to Replacement Therapies in Cardiogenic Shock: Insights From the Extracorporeal Life Support Organization. Circ. Heart Fail..

[34] Krasivskyi I., Grossmann C., Dechow M., Djordjevic I., Ivanov B., Gerfer S., Bennour W., Kuhn E., Sabashnikov A., Mader N. (2023). ECMO Retrieval Program: What Have We Learned So Far. Life.

[35] Hou J.Y., Li X., Yang S.G., Zheng J.L., Ma J.F., Su Y., Zhang Y.J., Guo K.F., Tu G.W., Luo Z. (2021). Veno-Arterial Extracorporeal Membrane Oxygenation for Patients Undergoing Heart Transplantation: A 7-Year Experience. Front. Med..

[36] Hou J.Y., Wang C.S., Lai H., Sun Y.X., Li X., Zheng J.L., Wang H., Luo J.C., Tu G.W., Luo Z. (2021). Veno-Arterial Extracorporeal Membrane Oxygenation for Patients Undergoing Acute Type A Aortic Dissection Surgery: A Six-Year Experience. Front. Cardiovasc. Med..

[37] Rob D., Spunda R., Lindner J., Rohn V., Kunstyr J., Balik M., Rulisek J., Kopecky P., Lips M., Smid O. (2017). A rationale for early extracorporeal membrane oxygenation in patients with postinfarction ventricular septal rupture complicated by cardiogenic shock. Eur. J. Heart Fail..

[38] Santise G., Panarello G., Ruperto C., Turrisi M., Pilato G., Giunta A., Sciacca S., Pilato M. (2014). Extracorporeal membrane oxygenation for graft failure after heart transplantation: A multidisciplinary approach to maximize weaning rate. Int. J. Artif. Organs.

[39] Flagiello M., Al Harthy A., Boccalini S., Jacquemet L., Obadia J.F., Baudry G., Pozzi M. (2021). Veno-arterial extracorporeal membrane oxygenation for COVID-19-associated acute myocardial injury complicated by refractory cardiogenic shock. J. Card. Surg..

[40] Popov A.F., Berger R., Schlensak C., Bongers M.N., Haeberle H., Acharya M., Lausberg H.F. (2020). Mechanical circulatory support for cardiovascular complications in a young COVID-19 patient. J. Card. Surg..

[41] Ling R.R., Ramanathan K., Poon W.H., Tan C.S., Brechot N., Brodie D., Combes A., MacLaren G. (2021). Venoarterial extracorporeal membrane oxygenation as mechanical circulatory support in adult septic shock: A systematic review and meta-analysis with individual participant data meta-regression analysis. Crit. Care.

[42] Chouairi F., Vallabhajosyula S., Mullan C., Mori M., Geirsson A., Desai N.R., Ahmad T., Miller P.E. (2020). Transition to Advanced Therapies in Elderly Patients Supported by Extracorporeal Membrane Oxygenation Therapy. J. Card. Fail..

[43] Lorusso R., Gelsomino S., Parise O., Mendiratta P., Prodhan P., Rycus P., MacLaren G., Brogan T.V., Chen Y.S., Maessen J. (2017). Venoarterial Extracorporeal Membrane Oxygenation for Refractory Cardiogenic Shock in Elderly Patients: Trends in Application and Outcome From the Extracorporeal Life Support Organization (ELSO) Registry. Ann. Thorac. Surg..

[44] Peigh G., Cavarocchi N., Keith S.W., Hirose H. (2015). Simple new risk score model for adult cardiac extracorporeal membrane oxygenation: Simple cardiac ECMO score. J. Surg. Res..

[45] Worku B., Khin S., Gaudino M., Avgerinos D., Gambardella I., D’Ayala M., Ramasubbu K., Gulkarov I., Salemi A. (2019). A Simple Scoring System to Predict Survival after Venoarterial Extracorporeal Membrane Oxygenation. J. Extra Corpor. Technol..

[46] Brener M.I., Rosenblum H.R., Burkhoff D. (2020). Pathophysiology and Advanced Hemodynamic Assessment of Cardiogenic Shock. Methodist Debakey Cardiovasc. J..

[47] Reynolds H.R., Hochman J.S. (2008). Cardiogenic shock: Current concepts and improving outcomes. Circulation.

[48] Standl T., Annecke T., Cascorbi I., Heller A.R., Sabashnikov A., Teske W. (2018). The Nomenclature, Definition and Distinction of Types of Shock. Dtsch. Arztebl. Int..

[49] Graf T., Desch S., Eitel I., Thiele H. (2015). Acute myocardial infarction and cardiogenic shock: Pharmacologic and mechanical hemodynamic support pathways. Coron. Artery Dis..

[50] Tehrani B.N., Truesdell A.G., Psotka M.A., Rosner C., Singh R., Sinha S.S., Damluji A.A., Batchelor W.B. (2020). A Standardized and Comprehensive Approach to the Management of Cardiogenic Shock. JACC Heart Fail..

[51] Berg D.D., Bohula E.A., van Diepen S., Katz J.N., Alviar C.L., Baird-Zars V.M., Barnett C.F., Barsness G.W., Burke J.A., Cremer P.C. (2019). Epidemiology of Shock in Contemporary Cardiac Intensive Care Units. Circ. Cardiovasc. Qual. Outcomes.

[52] Hussey P.T., von Mering G., Nanda N.C., Ahmed M.I., Addis D.R. (2022). Echocardiography for extracorporeal membrane oxygenation. Echocardiography.

[53] Doufle G., Roscoe A., Billia F., Fan E. (2015). Echocardiography for adult patients supported with extracorporeal membrane oxygenation. Crit. Care.

[54] Extracorporeal Life Support Organization (2017). Guidelines for Adult Respiratory Failure. https://www.elso.org/ecmo-resources/elso-ecmo-guidelines.aspx.

[55] Distelmaier K., Wiedemann D., Lampichler K., Toth D., Galli L., Haberl T., Steinlechner B., Heinz G., Laufer G., Lang I.M. (2020). Interdependence of VA-ECMO output, pulmonary congestion and outcome after cardiac surgery. Eur. J. Intern. Med..

[56] Grandin E.W., Nunez J.I., Willar B., Kennedy K., Rycus P., Tonna J.E., Kapur N.K., Shaefi S., Garan A.R. (2022). Mechanical Left Ventricular Unloading in Patients Undergoing Venoarterial Extracorporeal Membrane Oxygenation. J. Am. Coll. Cardiol..

[57] Nuding S., Werdan K. (2017). IABP plus ECMO-Is one and one more than two?. J. Thorac. Dis..

[58] Parissis H., Graham V., Lampridis S., Lau M., Hooks G., Mhandu P.C. (2016). IABP: History-evolution-pathophysiology-indications: What we need to know. J. Cardiothorac. Surg..

[59] Zeng P., Yang C., Chen J., Fan Z., Cai W., Huang Y., Xiang Z., Yang J., Zhang J., Yang J. (2022). Comparison of the Efficacy of ECMO With or Without IABP in Patients With Cardiogenic Shock: A Meta-Analysis. Front. Cardiovasc. Med..

[60] Glazier J.J., Kaki A. (2019). The Impella Device: Historical Background, Clinical Applications and Future Directions. Int. J. Angiol..

[61] Schrage B., Ibrahim K., Loehn T., Werner N., Sinning J.M., Pappalardo F., Pieri M., Skurk C., Lauten A., Landmesser U. (2019). Impella Support for Acute Myocardial Infarction Complicated by Cardiogenic Shock. Circulation.

[62] Fiorelli F., Panoulas V. (2021). Impella as unloading strategy during VA-ECMO: Systematic review and meta-analysis. Rev. Cardiovasc. Med..

[63] Mlcek M., Meani P., Cotza M., Kowalewski M., Raffa G.M., Kuriscak E., Popkova M., Pilato M., Arcadipane A., Ranucci M. (2021). Atrial Septostomy for Left Ventricular Unloading During Extracorporeal Membrane Oxygenation for Cardiogenic Shock: Animal Model. JACC Cardiovasc. Interv..

[64] Deshpande S.R., Kennedy K.F., Vincent R.N., Maher K.O. (2021). Atrial septostomy in patients supported with venoarterial extracorporeal membrane oxygenation: Analysis of the IMPACT registry data. Int. J. Artif. Organs.

[65] Loforte A., Baiocchi M., Dal Checco E., Gliozzi G., Fiorentino M., Lo Coco V., Martin Suarez S., Marrozzini C., Biffi M., Marinelli G. (2020). Percutaneous Pulmonary Artery Venting via Jugular Vein While on Peripheral Extracorporeal Life Support. ASAIO J..

[66] Loforte A., Baiocchi M., Gliozzi G., Coppola G., Di Bartolomeo R., Lorusso R. (2019). Percutaneous pulmonary artery venting via jugular vein while on peripheral extracorporeal membrane oxygenation running: A less invasive approach to provide full biventricular unloading. Ann. Cardiothorac. Surg..

[67] Phillip R., Howard J., Hawamdeh H., Tribble T., Gurley J., Saha S. (2023). Left Atrial Veno-Arterial Extracorporeal Membrane Oxygenation Case Series: A Single-Center Experience. J. Surg. Res..

[68] Zhang R., Kofidis T., Kamiya H., Shrestha M., Tessmann R., Haverich A., Klima U. (2006). Creatine kinase isoenzyme MB relative index as predictor of mortality on extracorporeal membrane oxygenation support for postcardiotomy cardiogenic shock in adult patients. Eur. J. Cardiothorac. Surg..

[69] Ohira S., Malekan R., Goldberg J.B., Lansman S.L., Spielvogel D., Kai M., Spencer P.J., Levine A., Pan S., Aggarwal-Gupta C. (2021). Axillary artery cannulation for veno-arterial extracorporeal membrane oxygenation support in cardiogenic shock. JTCVS Tech.

[70] Takayama H., Landes E., Truby L., Fujita K., Kirtane A.J., Mongero L., Yuzefpolskaya M., Colombo P.C., Jorde U.P., Kurlansky P.A. (2015). Feasibility of smaller arterial cannulas in venoarterial extracorporeal membrane oxygenation. J. Thorac. Cardiovasc. Surg..

[71] Kowalewski M., Malvindi P.G., Zielinski K., Martucci G., Slomka A., Suwalski P., Lorusso R., Meani P., Arcadipane A., Pilato M. (2020). Left Ventricle Unloading with Veno-Arterial Extracorporeal Membrane Oxygenation for Cardiogenic Shock. Systematic Review and Meta-Analysis. J. Clin. Med..

[72] Russo J.J., Aleksova N., Pitcher I., Couture E., Parlow S., Faraz M., Visintini S., Simard T., Di Santo P., Mathew R. (2019). Left Ventricular Unloading During Extracorporeal Membrane Oxygenation in Patients With Cardiogenic Shock. J. Am. Coll. Cardiol..

[73] Rupprecht L., Lunz D., Philipp A., Lubnow M., Schmid C. (2015). Pitfalls in percutaneous ECMO cannulation. Heart Lung Vessel.

[74] Wilson J., Fisher R., Caetano F., Soliman-Aboumarie H., Patel B., Ledot S., Price S., Vandenbriele C. (2022). Managing Harlequin Syndrome in VA-ECMO—Do not forget the right ventricle. Perfusion.

[75] Grant C., Richards J.B., Frakes M., Cohen J., Wilcox S.R. (2021). ECMO and Right Ventricular Failure: Review of the Literature. J. Intensive Care Med..

[76] Contento C., Battisti A., Agro B., De Marco M., Iaiza A., Pietraforte L., Pisani P., Proietti A., Vitalini E., Montalto A. (2020). A novel veno-arteriovenous extracorporeal membrane oxygenation with double pump for the treatment of Harlequin syndrome. Perfusion.

[77] Platts D.G., Sedgwick J.F., Burstow D.J., Mullany D.V., Fraser J.F. (2012). The role of echocardiography in the management of patients supported by extracorporeal membrane oxygenation. J. Am. Soc. Echocardiogr..

[78] Geller B.J., Sinha S.S., Kapur N.K., Bakitas M., Balsam L.B., Chikwe J., Klein D.G., Kochar A., Masri S.C., Sims D.B. (2022). Escalating and De-escalating Temporary Mechanical Circulatory Support in Cardiogenic Shock: A Scientific Statement From the American Heart Association. Circulation.

[79] Bhat A.G., Golchin A., Pasupula D.K., Hernandez-Montfort J.A. (2019). Right Sided Intracardiac Thrombosis during Veno-Arterial Extracorporeal Membrane Oxygenation: A Case Report and Literature Review. Case Rep. Crit. Care.

[80] Williams B., Bernstein W. (2016). Review of Venoarterial Extracorporeal Membrane Oxygenation and Development of Intracardiac Thrombosis in Adult Cardiothoracic Patients. J. Extra Corpor. Technol..

[81] Tan C., Rubenson D., Srivastava A., Mohan R., Smith M.R., Billick K., Bardarian S., Thomas Heywood J. (2017). Left ventricular outflow tract velocity time integral outperforms ejection fraction and Doppler-derived cardiac output for predicting outcomes in a select advanced heart failure cohort. Cardiovasc. Ultrasound.

[82] Vakil D., Soto C., D’Costa Z., Volk L., Kandasamy S., Iyer D., Ikegami H., Russo M.J., Lee L.Y., Lemaire A. (2021). Short-term and intermediate outcomes of cardiogenic shock and cardiac arrest patients supported by venoarterial extracorporeal membrane oxygenation. J. Cardiothorac. Surg..

[83] Rajsic S., Breitkopf R., Bukumiric Z., Treml B. (2022). ECMO Support in Refractory Cardiogenic Shock: Risk Factors for Mortality. J. Clin. Med..

[84] Rajsic S., Treml B., Jadzic D., Breitkopf R., Oberleitner C., Popovic Krneta M., Bukumiric Z. (2022). Extracorporeal membrane oxygenation for cardiogenic shock: A meta-analysis of mortality and complications. Ann. Intensive Care.

[85] Alhussein M., Moayedi Y., Posada J.D., Ross H., Hickey E., Rao V., Billia F. (2017). Ventricular Thrombosis Post-Venoarterial Extracorporeal Membrane Oxygenation. Circ. Heart Fail..

[86] Olson S.R., Murphree C.R., Zonies D., Meyer A.D., McCarty O.J.T., Deloughery T.G., Shatzel J.J. (2021). Thrombosis and Bleeding in Extracorporeal Membrane Oxygenation (ECMO) Without Anticoagulation: A Systematic Review. ASAIO J..

[87] Rajsic S., Breitkopf R., Rugg C., Bukumiric Z., Reitbauer J., Treml B. (2023). Thrombotic Events Develop in 1 Out of 5 Patients Receiving ECMO Support: An 11-Year Referral Centre Experience. J. Clin. Med..

[88] Colman E., Yin E.B., Laine G., Chatterjee S., Saatee S., Herlihy J.P., Reyes M.A., Bracey A.W. (2019). Evaluation of a heparin monitoring protocol for extracorporeal membrane oxygenation and review of the literature. J. Thorac. Dis..

[89] Rajsic S., Breitkopf R., Jadzic D., Popovic Krneta M., Tauber H., Treml B. (2022). Anticoagulation Strategies during Extracorporeal Membrane Oxygenation: A Narrative Review. J. Clin. Med..

[90] Rajsic S., Treml B., Jadzic D., Breitkopf R., Oberleitner C., Bachler M., Bosch J., Bukumiric Z. (2023). aPTT-guided anticoagulation monitoring during ECMO support: A systematic review and meta-analysis. J. Crit. Care.

[91] Rajsic S., Breitkopf R., Treml B., Jadzic D., Oberleitner C., Oezpeker U.C., Innerhofer N., Bukumiric Z. (2023). Association of aPTT-Guided Anticoagulation Monitoring with Thromboembolic Events in Patients Receiving V-A ECMO Support: A Systematic Review and Meta-Analysis. J. Clin. Med..

[92] Wood K.L., Ayers B., Gosev I., Kumar N., Melvin A.L., Barrus B., Prasad S. (2020). Venoarterial-Extracorporeal Membrane Oxygenation Without Routine Systemic Anticoagulation Decreases Adverse Events. Ann. Thorac. Surg..

[93] Fang Z.A., Navaei A.H., Hensch L., Hui S.R., Teruya J. (2020). Hemostatic Management of Extracorporeal Circuits Including Cardiopulmonary Bypass and Extracorporeal Membrane Oxygenation. Semin. Thromb. Hemost..

[94] Thomas J., Kostousov V., Teruya J. (2018). Bleeding and Thrombotic Complications in the Use of Extracorporeal Membrane Oxygenation. Semin. Thromb. Hemost..

[95] Ohira S., Kawamura M., Ahern K., Cavarocchi N., Hirose H. (2020). Aggressive placement of distal limb perfusion catheter in venoarterial extracorporeal membrane oxygenation. Int. J. Artif. Organs.

[96] Ohira S., Pan S., Levine A., Aggarwal-Gupta C., Lanier G.M., Gass A.L., Spielvogel D., Kai M. (2023). Simple technique of distal leg perfusion during heart transplant in patients with preoperative veno-arterial extracorporeal membrane oxygenation support. Perfusion.

[97] Patton-Rivera K., Beck J., Fung K., Chan C., Beck M., Takayama H., Takeda K. (2018). Using near-infrared reflectance spectroscopy (NIRS) to assess distal-limb perfusion on venoarterial (V-A) extracorporeal membrane oxygenation (ECMO) patients with femoral cannulation. Perfusion.

[98] Tanaka D., Hirose H., Cavarocchi N., Entwistle J.W. (2016). The Impact of Vascular Complications on Survival of Patients on Venoarterial Extracorporeal Membrane Oxygenation. Ann. Thorac. Surg..

[99] Wong J.K., Smith T.N., Pitcher H.T., Hirose H., Cavarocchi N.C. (2012). Cerebral and lower limb near-infrared spectroscopy in adults on extracorporeal membrane oxygenation. Artif. Organs.

[100] Askenazi D.J., Selewski D.T., Paden M.L., Cooper D.S., Bridges B.C., Zappitelli M., Fleming G.M. (2012). Renal replacement therapy in critically ill patients receiving extracorporeal membrane oxygenation. Clin. J. Am. Soc. Nephrol..

[101] Selewski D.T., Wille K.M. (2021). Continuous renal replacement therapy in patients treated with extracorporeal membrane oxygenation. Semin. Dial..

[102] Kilburn D.J., Shekar K., Fraser J.F. (2016). The Complex Relationship of Extracorporeal Membrane Oxygenation and Acute Kidney Injury: Causation or Association?. BioMed Res. Int..

[103] Ostermann M., Connor M., Kashani K. (2018). Continuous renal replacement therapy during extracorporeal membrane oxygenation: Why, when and how?. Curr. Opin. Crit. Care.

[104] Aboul Nour H., Poyiadji N., Mohamed G., Alsrouji O.K., Ramadan A.R., Griffith B., Marin H., Chebl A.B. (2021). Challenges of acute phase neuroimaging in VA-ECMO, pitfalls and alternative imaging options. Interv. Neuroradiol..

[105] Al-Kawaz M., Shou B., Prokupets R., Whitman G., Geocadin R., Cho S.M. (2022). Mild hypothermia and neurologic outcomes in patients undergoing venoarterial extracorporeal membrane oxygenation. J. Card. Surg..

[106] Pillai A.K., Bhatti Z., Bosserman A.J., Mathew M.C., Vaidehi K., Kalva S.P. (2018). Management of vascular complications of extra-corporeal membrane oxygenation. Cardiovasc. Diagn. Ther..

[107] Burgos L.M., Seoane L., Diez M., Baro Vila R.C., Furmento J.F., Vrancic M., Aissaoui N. (2023). Multiparameters associated to successful weaning from VA ECMO in adult patients with cardiogenic shock or cardiac arrest: Systematic review and meta-analysis. Ann. Card. Anaesth..

[108] Cavarocchi N.C., Pitcher H.T., Yang Q., Karbowski P., Miessau J., Hastings H.M., Hirose H. (2013). Weaning of extracorporeal membrane oxygenation using continuous hemodynamic transesophageal echocardiography. J. Thorac. Cardiovasc. Surg..

[109] Aissaoui N., El-Banayosy A., Combes A. (2015). How to wean a patient from veno-arterial extracorporeal membrane oxygenation. Intensive Care Med..

[110] Lusebrink E., Stremmel C., Stark K., Joskowiak D., Czermak T., Born F., Kupka D., Scherer C., Orban M., Petzold T. (2020). Update on Weaning from Veno-Arterial Extracorporeal Membrane Oxygenation. J. Clin. Med..

[111] Pappalardo F., Pieri M., Arnaez Corada B., Ajello S., Melisurgo G., De Bonis M., Zangrillo A. (2015). Timing and Strategy for Weaning From Venoarterial ECMO are Complex Issues. J. Cardiothorac. Vasc. Anesth..

[112] Ortuno S., Delmas C., Diehl J.L., Bailleul C., Lancelot A., Naili M., Cholley B., Pirracchio R., Aissaoui N. (2019). Weaning from veno-arterial extra-corporeal membrane oxygenation: Which strategy to use?. Ann. Cardiothorac. Surg..

[113] Firstenberg M.S., Orsinelli D.A. (2012). ECMO and ECHO: The evolving role of quantitative echocardiography in the management of patients requiring extracorporeal membrane oxygenation. J. Am. Soc. Echocardiogr..

[114] Loforte A., Marinelli G., Musumeci F., Folesani G., Pilato E., Martin Suarez S., Montalto A., Lilla Della Monica P., Grigioni F., Frascaroli G. (2014). Extracorporeal membrane oxygenation support in refractory cardiogenic shock: Treatment strategies and analysis of risk factors. Artif. Organs.

[115] Negi S.I., Sokolovic M., Koifman E., Kiramijyan S., Torguson R., Lindsay J., Ben-Dor I., Suddath W., Pichard A., Satler L. (2016). Contemporary Use of Veno-Arterial Extracorporeal Membrane Oxygenation for Refractory Cardiogenic Shock in Acute Coronary Syndrome. J. Invasive Cardiol..

[116] Khorsandi M., Dougherty S., Bouamra O., Pai V., Curry P., Tsui S., Clark S., Westaby S., Al-Attar N., Zamvar V. (2017). Extra-corporeal membrane oxygenation for refractory cardiogenic shock after adult cardiac surgery: A systematic review and meta-analysis. J. Cardiothorac. Surg..

[117] Garcia-Gigorro R., Renes-Carreno E., Perez-Vela J.L., Marin-Mateos H., Gutierrez J., Corres-Peiretti M.A., Delgado J.F., Perez-de la Sota E., Cortina-Romero J.M., Montejo-Gonzalez J.C. (2017). Mechanical support with venoarterial extracorporeal membrane oxygenation (ECMO-VA): Short-term and long-term prognosis after a successful weaning. Med. Intensiv..

[118] Fux T., Holm M., Corbascio M., Lund L.H., van der Linden J. (2019). VA-ECMO Support in Nonsurgical Patients With Refractory Cardiogenic Shock: Pre-Implant Outcome Predictors. Artif. Organs.

[119] Krasivskyi I., Ivanov B., Vehrenberg J., Eghbalzadeh K., Gerfer S., Gaisendrees C., Kuhn E., Sabashnikov A., Mader N., Djordjevic I. (2022). Sex-Related Differences in Short-Term Outcomes after Mobile VA-ECMO Implantation: Five-Year Experience of an ECMO Retrieval Program. Life.

[120] Ostadal P., Rokyta R., Karasek J., Kruger A., Vondrakova D., Janotka M., Naar J., Smalcova J., Hubatova M., Hromadka M. (2023). Extracorporeal Membrane Oxygenation in the Therapy of Cardiogenic Shock: Results of the ECMO-CS Randomized Clinical Trial. Circulation.

[121] Banning A.S., Sabate M., Orban M., Gracey J., López-Sobrino T., Massberg S., Kastrati A., Bogaerts K., Adriaenssens T., Berry C. (2023). Venoarterial extracorporeal membrane oxygenation or standard care in patients with cardiogenic shock complicating acute myocardial infarction: The multicentre, randomised EURO SHOCK trial. EuroIntervention.

[122] Abrams D., MacLaren G., Lorusso R., Price S., Yannopoulos D., Vercaemst L., Belohlavek J., Taccone F.S., Aissaoui N., Shekar K. (2022). Extracorporeal cardiopulmonary resuscitation in adults: Evidence and implications. Intensive Care Med..

[123] Rob D., Smalcova J., Smid O., Kral A., Kovarnik T., Zemanek D., Kavalkova P., Huptych M., Komarek A., Franek O. (2022). Extracorporeal versus conventional cardiopulmonary resuscitation for refractory out-of-hospital cardiac arrest: A secondary analysis of the Prague OHCA trial. Crit. Care.

[124] Kim S.J., Kim H.J., Lee H.Y., Ahn H.S., Lee S.W. (2016). Comparing extracorporeal cardiopulmonary resuscitation with conventional cardiopulmonary resuscitation: A meta-analysis. Resuscitation.

[125] Tonna J.E., Johnson N.J., Greenwood J., Gaieski D.F., Shinar Z., Bellezo J.M., Becker L., Shah A.P., Youngquist S.T., Mallin M.P. (2016). Practice characteristics of Emergency Department extracorporeal cardiopulmonary resuscitation (eCPR) programs in the United States: The current state of the art of Emergency Department extracorporeal membrane oxygenation (ED ECMO). Resuscitation.

[126] Chen Y.S., Lin J.W., Yu H.Y., Ko W.J., Jerng J.S., Chang W.T., Chen W.J., Huang S.C., Chi N.H., Wang C.H. (2008). Cardiopulmonary resuscitation with assisted extracorporeal life-support versus conventional cardiopulmonary resuscitation in adults with in-hospital cardiac arrest: An observational study and propensity analysis. Lancet.

[127] Belohlavek J., Smalcova J., Rob D., Franek O., Smid O., Pokorna M., Horak J., Mrazek V., Kovarnik T., Zemanek D. (2022). Effect of Intra-arrest Transport, Extracorporeal Cardiopulmonary Resuscitation, and Immediate Invasive Assessment and Treatment on Functional Neurologic Outcome in Refractory Out-of-Hospital Cardiac Arrest: A Randomized Clinical Trial. JAMA.

[128] Shin T.G., Jo I.J., Sim M.S., Song Y.B., Yang J.H., Hahn J.Y., Choi S.H., Gwon H.C., Jeon E.S., Sung K. (2013). Two-year survival and neurological outcome of in-hospital cardiac arrest patients rescued by extracorporeal cardiopulmonary resuscitation. Int. J. Cardiol..

[129] Tonna J.E., Selzman C.H., Girotra S., Presson A.P., Thiagarajan R.R., Becker L.B., Zhang C., Rycus P., Keenan H.T., American Heart Association Get With the Guidelines-Resuscitation, I (2022). Resuscitation Using ECPR During In-Hospital Cardiac Arrest (RESCUE-IHCA) Mortality Prediction Score and External Validation. JACC Cardiovasc. Interv..

[130] Ostadal P., Rokyta R., Kruger A., Vondrakova D., Janotka M., Smid O., Smalcova J., Hromadka M., Linhart A., Belohlavek J. (2017). Extra corporeal membrane oxygenation in the therapy of cardiogenic shock (ECMO-CS): Rationale and design of the multicenter randomized trial. Eur. J. Heart Fail..

